# Cannabidiol and Intestinal Motility: a Systematic Review

**DOI:** 10.1016/j.cdnut.2023.101972

**Published:** 2023-07-17

**Authors:** Galaxie Story, Carrie-Ellen Briere, D. Julian McClements, David A. Sela

**Affiliations:** 1 Department of Food Science, University of Massachusetts, Amherst, MA, United States; 2 Elaine Marieb College of Nursing, University of Massachusetts, Amherst, MA, United States; 3 Department of Nutrition, University of Massachusetts, Amherst, MA, United States; 4 Department of Microbiology and Physiological Systems, University of Massachusetts Medical School, Worcester, MA, United States

**Keywords:** cannabidiol, intestinal motility, cannabinoids, gastrointestinal, irritable bowel syndrome, inflammatory bowel disease, functional foods, plant bioactive

## Abstract

Cannabidiol (CBD) is a non-intoxicating cannabinoid extracted from the cannabis plant that is used for medicinal purposes. Ingestion of CBD is claimed to address several pathologies, including gastrointestinal disorders, although limited evidence has been generated thus far to substantiate many of its health claims. Nevertheless, CBD usage as an over-the-counter treatment for gastrointestinal disorders is likely to expand in response to increasing commercial availability, permissive legal status, and acceptance by consumers. This systematic review critically evaluates the knowledge boundaries of the published research on CBD, intestinal motility, and intestinal motility disorders. Research on CBD and intestinal motility is currently limited but does support the safety and efficacy of CBD for several therapeutic applications, including seizure disorders, inflammatory responses, and upper gastrointestinal dysfunction (i.e., nausea and vomiting). CBD, therefore, may have therapeutic potential for addressing functional gastrointestinal disorders. The results of this review show promising *in vitro* and preclinical data supporting a role of CBD in intestinal motility. This includes improved gastrointestinal-related outcomes in murine models of colitis. These studies, however, vary by dose, delivery method, and CBD-extract composition. Clinical trials have yet to find a conclusive benefit of CBD on intestinal motility disorders, but these trials have been limited in scope. In addition, critical factors such as CBD dosing parameters have not yet been established. Further research will establish the efficacy of CBD in applications to address intestinal motility.

## Introduction

**FIGURE 1:**
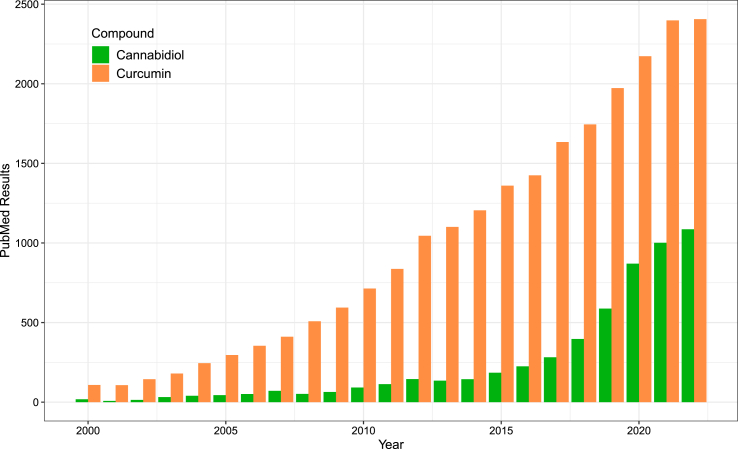
Comparison of PubMed search results from 2000 to 2022 for the terms “cannabidiol” and “curcumin.”

Cannabis has been used for its medicinal properties for centuries [1], and research on the mechanism of action and physiological effects of specific cannabinoids have been conducted over the past few decades. Among cannabinoids, cannabidiol (CBD) has been more extensively studied, although somewhat limited in scope relative to other phytochemical bioactives (Figure 1). It was not until 2011 that PubMed results first exceeded 100 citations per year for the search term “cannabidiol,” and the volume of articles written on this topic increased to 1008 by 2021 [2].

CBD is a non-intoxicating lipid-soluble phytocannabinoid that is incorporated into popular supplements due to its purported health benefits [3]. Fundamental CBD biological activity has been studied mechanistically as well as in clinical studies to evaluate its impact on psychological disorders, neurological disorders, cancers, and gastrointestinal diseases [4]. Data supporting the anti-seizure effects of CBD provide the most well-established evidence of a therapeutic role for CBD [5]. In addition, studies report that CBD alleviates chemotherapy-induced nausea and vomiting, has analgesic and anxiolytic effects, and reduces withdrawal and craving in patients with substance use disorder [[6], [7], [8]]. A 2019 survey reported 14% of US citizens use CBD products to address several issues, with most respondents using CBD to treat pain, anxiety, sleep, or arthritis [9]. There is an accumulation of scientific evidence for CBD efficacy in treating these ailments, although more studies are required to standardize optimal dosing and delivery. Nevertheless, there is consistent evidence for CBD improving clinical outcomes in anxiety disorders in preclinical models, with a growing body of evidence of anxiolytic properties in humans with limited side effects [10]. This anxiolytic effect may be related to other benefits such as improving sleep quality. Furthermore, there is evidence for CBD-mediated pain relief and treatment of arthritis [11,12]. Due to the many unknowns regarding CBD human subject research (i.e., dose, delivery vehicle, etc.), standardized study protocols have yet to be established or implemented, which makes cross-study comparisons difficult.

Given the evidence that CBD mitigates chemotherapy-induced nausea and vomiting, the extent to which CBD improves bowel dysfunction is of broad interest and may contribute to consumer enthusiasm for CBD. Other cannabinoids, such as an enantiomer of Δ9-tetrahydrocannabinol (THC), dronabinol, have been demonstrated to improve measures of fasting colonic motility in participants with diarrhea-predominant irritable bowel syndrome (IBS) [13]. These results were inconsistent in a longer-term follow-up study [14] that did not indicate bowel motility improvement. Moreover, dronabinol promotes an intoxicating effect, which reduces consumer acceptability and application [15].

In addition to supplements and food/beverages that incorporate CBD, a drug containing purified CBD, Epidiolex, intended to treat rare forms of epilepsy was approved by the US FDA in 2018. Subsequently, and with increasing over-the-counter availability, cannabidiol was removed from the federal list of controlled substances by the US Drug Enforcement Administration after previous classification as a Schedule V substance [16].

CBD is not associated with major safety risks, although the US FDA has declined to confer “Generally Recognized as Safe” status, citing knowledge gaps regarding toxicity. Thus, this ambiguous position lags behind public consumption, similar to the need to increase the scientific understanding supporting cannabinoid efficacy. Regardless, CBD is easily available and somewhat ubiquitously entrenched in the US food system as it is sold in the form of candies, tinctures, and beverages. The global revenue for CBD sales is forecasted to increase to over $5.3 billion by 2025, $3.4 billion of that being in North America [17].

As with many clinical studies that are conducted with much higher concentrations of purified substances than typically consumed, some mild to moderate adverse events were reported for a minority of participants. This includes changes in somnolence, decreased appetite, diarrhea, hormone changes, decreased fertility, and hepatic impairment; detailed accounts and analysis of CBD-associated adverse events have been reviewed previously [[18], [19], [20]]. Many of these side effects have been demonstrated at higher dosing (>200 mg/kg body mass/d) which far exceeds typical oral delivery methods (e.g., 5–20 mg gummy) and exceeds current maximum recommendations (20 mg/kg body mass/d). A 2019 study reported no serious adverse effects of CBD administration in participants with underlying hepatic impairment, indicating that CBD use is generally tolerated [21].

The understanding of the absorption, metabolism, excretion, and effects of CBD remain incomplete due, in part, to multiple interactions between CBD and human physiology. There is potential for drug-drug interactions, and thus, medical supervision of CBD administration is justified in at-risk populations as is generally recommended with supplements [22,23]. Comprehensive reviews of pharmacokinetic studies including the metabolic fate of cannabinoids have been performed and are reviewed elsewhere [[24], [25], [26]].

### The endocannabinoid system

The endocannabinoid system (ECS) includes endocannabinoid receptors, endogenous ligands (eg, anandamide and 2-arachidonoylglycerol), and downstream metabolic products. Endocannabinoid receptors are found throughout the human body, including the central nervous, immune, gastrointestinal, and respiratory systems. The exact physiological role of the ECS is currently being investigated, although it has been implicated in immune, metabolic, and nervous system homeostasis in addition to having a regulatory role via the gut-brain axis [[27], [28], [29], [30], [31]].

CB1 and CB2 receptors are the best characterized G protein-coupled receptors in the ECS, and CBD interacts with these receptors at low affinity [[32], [33], [34], [35], [36]]. Interestingly, CB1 receptors are found in the enteric nervous system, specifically in the myenteric plexus, which controls intestinal motility and is the focus of several studies of IBS [37,38]. Polymorphisms in the CB1 gene (*CNR1*) have been linked with IBS [14,39,40]. CB2 receptors, in contrast, are more abundant in immune cells and have been implicated in modulating inflammatory responses [36]. In addition, G protein-coupled receptor 55 (GPR55) has been linked to the ECS [41]. This receptor may potentiate actions such as regulation of inflammation, pain, neurological function, cancer cell proliferation, as well as gastrointestinal motility [[41], [42], [43]]. GPR55 expression is most abundant in the adrenal glands, jejunum, ileum, and parts of the central nervous system [42].

Fatty acid amide hydrolase (FAAH) degrades endocannabinoids, including 2-arachidonoylglycerol and anandamide [44]. Cannabinoid receptors (e.g., CB1) experience increased activation with FAAH inhibition [45,46]. Interestingly, CBD inhibits FAAH to potentially increase levels of endocannabinoids, which in turn act on endocannabinoid receptors [47]. FAAH is expressed in both the small and large intestine and has been postulated to contribute to gastrointestinal motility and homeostasis [48]. A comprehensive review on the role of the ECS and gastrointestinal motility has been extensively reviewed elsewhere [[48], [49], [50]].

CBD may also interact with nonendocannabinoid receptors involved in gastrointestinal function, such as those for 5-hydroxytryptamine (5-HT), also referred to as serotonin. 5-HT receptors are found throughout the gastrointestinal tract and modulate gut motility [51,52]. The effect of 5-HT on gastrointestinal function has been well established, and pharmaceuticals acting on 5-HT receptors are used to treat functional gastrointestinal disorders [51,53]. The effects of CBD on all subtypes of 5-HT receptors have yet to be reported as research has focused primarily on interactions with the 5-HT1A receptor. CBD activation of 5-HT1A receptors has been implicated in its antidepressant, antianxiolytic, antiemetic, and antinausea effects [[54], [55], [56], [57]]. Finally, it has also been proposed that CBD activates peroxisome proliferator-activated receptor-γ (PPAR-γ), a receptor currently understood to function external to the ECS and is involved in the regulation of gastrointestinal and neurological homeostasis. Additional research is required to fully characterize this interaction; however, preclinical and human biopsy data have demonstrated CBD activates peroxisome proliferator-activated receptor-γ while exerting neuroprotective and anti-inflammatory effects [58,59].

### Intestinal motility and motility disorders

Intestinal motility is controlled through smooth muscle contractions induced by the enteric and central nervous system [52]. These contractions move the contents of the intestines through the digestive tract and are controlled through neurohumoral, electric, and cellular mechanisms. This induces localized segmenting movements and powerful contractile waves, known as mass peristalsis [52,60]. Hormones, including insulin, cholecystokinin, and gastrin are involved in intestinal motility in addition to neurotransmitters (e.g., serotonin and acetylcholine) [52]. Irregularities in intestinal motility are often associated with inflammatory processes, such as during colitis, that could impact multiple aspects of the system.

IBS is a functional gastrointestinal disorder characterized by pain and changes in stool frequency, which can be subtyped into diarrhea (hypermotility), constipation (hypomotility), or mixed predominance [61]. It is estimated that the prevalence of IBS in the general population is ∼5% to 10% [61,62]. The pathophysiology of IBS remains poorly defined, and its etiology is likely multifactorial [63]. Several studies have investigated the role of subclinical inflammation as part of its etiology [64,65].

Inflammatory bowel disease (IBD) is a chronic inflammatory disorder that is either localized in the colon and rectum (ulcerative colitis) or impacts the entire gastrointestinal tract (Crohn’s disease). A common symptom of both forms of IBD is diarrhea and clinical inflammation [66]. Altered gastrointestinal motility and functional motility disorders can be present in patients with IBD and often overlap or are confused with symptoms secondary to inflammation [62,66].

CBD may mitigate upper gastrointestinal dysfunction, such as nausea and vomiting, and interacts with a variety of receptors implicated in intestinal motility. This is in addition to the anxiolytic properties of CBD, which may be helpful in addressing IBS, which is associated with elevated anxiety [67]. Accordingly, selective serotonin reuptake inhibitors used to treat anxiety have exhibited efficacy in addressing IBS, most notably constipation-predominant IBS, by increasing motility. Additional pharmaceuticals, such as alosetron, treat diarrhea-predominant IBS by targeting 5-HT receptors, and it is possible that CBD interactions with ECS receptors may be a therapeutic option in IBS and other functional gastrointestinal tract disorders related to intestinal motility. This review summarizes current research on the use of CBD in intestinal motility as well as identifies knowledge gaps for future research.

## Methods

### Eligibility criteria

Studies in which CBD was administered, applied, or otherwise utilized *in vitro* or *in vivo* with the aim of studying intestinal motility were included. This comprises purified CBD and full spectrum extract, which may contain other cannabinoids, terpenes, and flavonoids in lesser concentrations than CBD. Studies were excluded that used CBD without any measures of intestinal motility as well as studies of other cannabinoids, including the synthetic cannabinoid and structural isomer of CBD often referred to as “abnormal CBD.” Published primary literature was included, and no publication date restriction was imposed. Case reports and review articles were excluded. PubMed and ClinicalTrials.gov databases were searched from inception to December 14, 2021, and the reference lists of the identified articles were reviewed. The databases were searched again on December 7, 2022 to identify any additional articles.

### Search

The following search terms were used in PubMed and the ClinicalTrials.gov databases: cannabidiol; CBD; motility; intestine; inflammatory bowel disease; IBD; IBS; irritable bowel syndrome; colitis; colon; colorectal; intestinal inflammation; gut; microbiome; and microbiota. See Supplemental Material 1 for an exhaustive list of search terms. Databases were searched from inception to December 14, 2021 and the search was updated on December 7, 2022.

### Study selection

The online article search application, Rayyan, was utilized to manage search results [68]. After database searching, all article titles and abstracts were reviewed, and those that clearly did not address the review purpose and meet the a priori inclusion criteria were excluded from further review. The remaining articles were subjected to a full text review to determine inclusion eligibility.

### Intestinal motility measures

Lack of standardization in methods and outcome measures CBD research limited the specificity of intestinal motility measures defined by the authors. Stool frequency and consistency, along with disease activity scores aimed at assessing intestinal function, meal passage rates/transit, and measures of intestine membrane potential and contractile forces were included in the definition of intestinal motility measurements.

### Data extraction

Information from included articles was chosen for its relevance to CBD and intestinal motility as specified in the review purpose and inclusion/exclusion criteria.

### Risk of bias

Due to the relative increase in recent CBD research and nonuniformity in the field overall, bias was unable to be assessed across studies. Moreover, the majority of CBD research has thus far been conducted with *in vitro* or animal models. It is generally understood that these models may not be fully predictive of human physiological responses.

## Results

### Article search results

**FIGURE 2:**
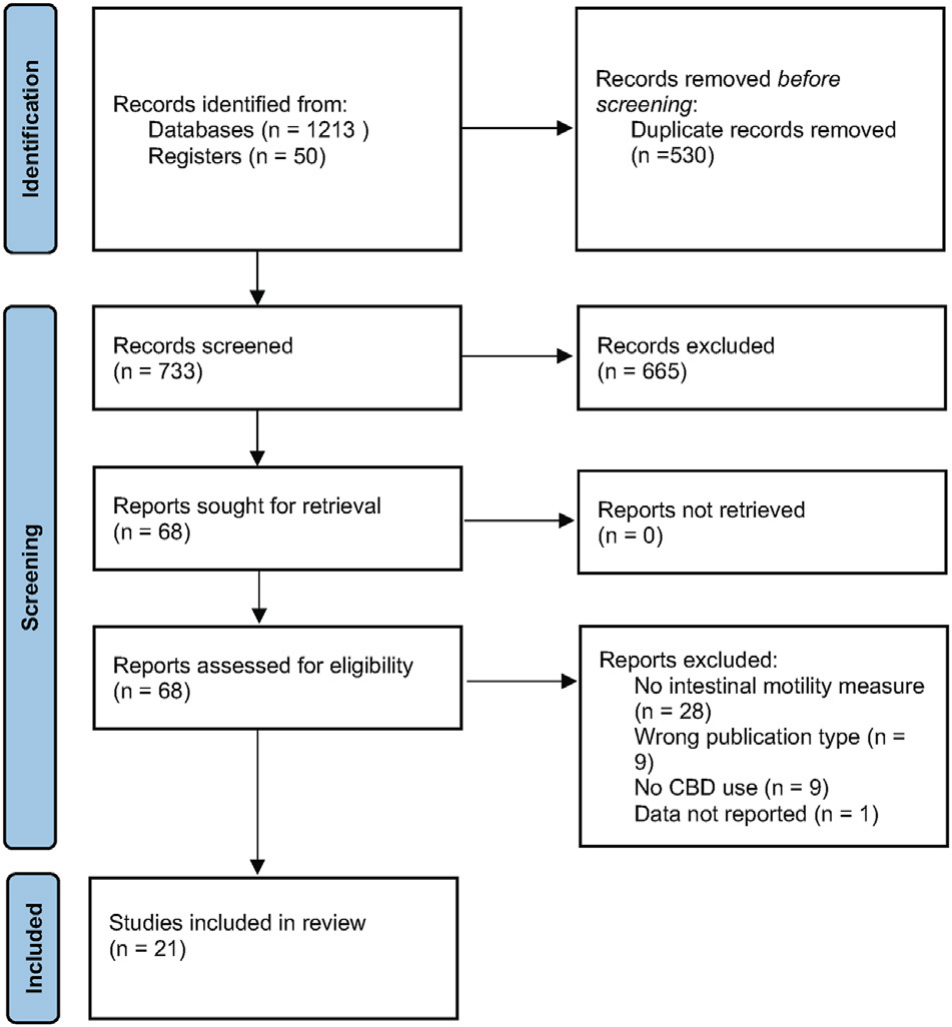
Preferred Reporting for Systematic Reviews and Meta-Analyses (PRISMA) flow diagram.

A PRISMA diagram displaying the flow of articles through the selection process is shown in Figure 2. The initial search identified 1263 results, which included 530 duplicates. After title and abstract screening, 68 articles remained for full text review. Forty-seven articles were excluded due to lack of motility measurements, CBD was not used, excluded publication type, or the data were not reported. After full text examination, 21 articles remained to be reviewed fully. Articles that met criteria and were fully examined are summarized in Table 1.

**TABLE 1:** Characteristics and findings of the effect of CBD on intestinal motility.

Study	Design	Motility Assessment	Additional Treatment	CBD dose, route	Effects on Motility Related Parameters	Summary
Cluny et al., 2011 [88]	*in vitro/ex vivo*	Resting tissue tension (grams tension*, S. murinus* whole intestine) Carbachol and EFS contractile response (grams tension*, S. murinus* whole intestine) KCl response (grams tension, S. murinus whole intestine)	AM251 AM630 TTX	10 nM–30 μM, organ bath	**Carbachol response reduction (% reduction):** CBD (3 μM) + carbachol (10 μM, 30 μM) on proximal intestine: 14.3 ± 3.3∗, 13.9 ± 3.3∗, respectively CBD (10 μM) + carbachol treated proximal and central intestine: contractions ↓ significantly∗ CBD (10 μM) + carbachol on terminal intestine: ↓ contractions significantly∗ **EFS:** CBD (10 μM) at 4–20 Hz on proximal & central intestine: ↓ contractions significantly (4 Hz∗, 10 Hz∗, and 20 Hz∗∗) **KCl response:** CBD (10 μM) decreased contraction response to KCl in all parts of the intestine∗∗∗ **CBD + TTX or CB antagonist response:** TTX and the CB antagonists did not modify the effect of CBD	CBD ↓ resting tissue tension in all parts of the intestine CBD ↓ contraction response to carbachol & EFS CBD ↓ contraction response to KCl AM251, AM630, & TTX did not modify effect of CBD
Jamontt et al., 2010 [90]	*in vitro/ex vivo*	Spontaneous activity (amplitude & duration, Charles River Wistar rat colon segments) Carbachol response; EFS contractile response (potency and duration; amplitude, Charles River Wistar rat colon segments)	Carbachol THC TNBS	5–20 mg/kg, i.p	**Spontaneous contractions, amplitude (gram/gram dry tissue weight); duration (seconds):** Control (no TNBS tx): 195 ± 19; 26.9 ± 1.7 Vehicle (TNBS tx): 37 ± 5; 62.8 ± 4.5 CBD 10 mg/kg: 67 ± 11∗; 46.2 ± 6.5∗ CBD 20 mg/kg: 55 ± 10; 38.7 ± 2.8∗ CBD (10 mg/kg) + THC (5 mg/kg): 66 ± 10; 46.3 ± 3.4∗ CBD (10 mg/kg) + THC (10 mg/kg): 89 ± 9∗; 44.5 ± 5.6∗ **Carbachol response, potency (LogEC50); max contraction (gram/gram dry tissue weight):** Control: 6.76 ± 0.24; 329 ± 35 Vehicle: 7.06 ± 0.30; 107 ± 11 CBD (10 mg/kg): 6.52 ± 0.39; 230 ± 45∗ CBD (10 mg/kg) + THC (10 mg/kg): data reported in figure∗ **EFS:** CBD alone no significant effect CBD (10 mg/kg) + THC (10 mg/kg): data reported in figure∗	TBNS ↓ amplitude & duration of spontaneous contractions in rat colon strips & carbachol response CBD ↑ amplitude & duration of spontaneous contractions in colon strips from TNBS treated rats CBD + THC ↑ amplitude & duration of SC in colon strips from TNBS treated rats CBD ↑ contractions to carbachol in colon strips from TNBS treated rats CBD did not impact the tissue response to EFS; CBD + THC had a significant effect CBD doses followed a bell-shaped activity curve & CBD + THC demonstrated an additive effect
Li, 2013 [76]	*in vitro/ex vivo* *& in vivo*	Evans blue solution (whole gut transit %, Charles River CD1 mice) Time to colonic bead expulsion (% of control, Charles River CD1 mice) EFS contraction response (Charles River CD1 mouse ileum & colon segments)	*In vivo:* O-1602, WIN55,212-2 In vitro: O-1602, WIN55,212-2	*In vivo:* 0.5 mg/kg, i.p; 20 μg, i.c.v. *In vitro:* 0.001–0.1 μM	**Whole gut transit (% of control), *in vivo*:** CBD (0.5 mg/kg, IP): Counteracted effect of O-1602∗ CBD (20 μg ICV): Counteracted effect of O-1602∗ and WIN55,212-2∗ **Time to bead expulsion (% of control), *in vivo*:** CBD (0.5 mg/kg, IP) + O-1602: significantly decreased compared to O-1602 group∗ **EFS contraction response, *in vitro*:** CBD (0.1 μM) (colon): 37.6 ± 5.7%∗ CBD (0.001–0.01 μM) + O-1602 (ileum): CBD blocked the inhibitory effect of O-1602 (data reported in figure)∗ CBD (0.01 μM) + O-1602 (colon): CBD blocked the inhibitory effect of O-1602 (data reported in figure)∗	O-1602 and WIN55,212-2 ↓ whole gut transit and colonic bead expulsion CBD alone had no effect on whole gut transit CBD blocked effect of O-1602 on whole gut transit and bead expulsion CBD ↓ EFS contraction response in colon at the highest dose tested CBD blocked the effect of O-1602 in the colon and ileum
De Filippis et al., 2008 [75]	*in vitro/ex vivo* & *in vivo*	Glass bead transit (GC, swiss OF1 mice) Contractile response (swiss OF1 mouse jejunum)	*In vivo*: AM251, LPS *In vitro:* Capsaicin	*In vivo:* 10 mg/kg, i.p. *In vitro:* 0.01–10 μM	**Geometric center (GC), *in vivo*:** Control (no LPS): CBD did not have a significant effect on the GC LPS: Significant reduction in GC compared to control∗ LPS + CBD: Significant reduction in GC compared to control∗ and LPS alone∗ LPS + CBD + AM251: Significant reduction in GC compared to control∗ **Contraction response, *in vitro*:** CBD did not induce any contraction.	GC was calculated as Σ (%beads per segment x segment number)/100 LPS ↓ geometric center (GC) CBD had no effect on GC of control mice CBD further reduced the GC of LPS treated mice CBD failed to induce contractions in mouse jejunal segments LPS significantly increased FAAH expression CBD significantly reduced FAAH expression in LPS treated mice
Chester et al., 1973 [69]	*in vivo*	Charcoal meal passage (& transit, SW mice)	N/A	6–30 mg/kg, oral gavage	Inactive all doses, values not reported	CBD did not affect charcoal meal passage rate
Anderson et al., 1974 [71]	*in vivo*	Charcoal meal passage rate (% transit of control, SW mice)	THC Cannabinol	0–50 mg/kg, oral gavage	*Passage rate (distance traveled % of control):* Control: 100 ± 2.9 CBD 10 mg/kg: 82.8 ± 2.4∗ significantly less than control CBD + THC (10 mg/kg each): 48.5 ± 2.4∗ significantly less than CBD + cannabinol (10 mg/kg each) and THC + cannabinol (10 mg/kg each), significantly more than THC + CBD (10 mg/kg and 40 mg/kg respectively)	CBD ↓ the % transit of a charcoal meal CBD followed a bell-shaped activity curve Additional cannabinoid interactions were identified such as those between THC, cannabinol, and cannabidiol on % transit.
Sabo et al., 2013 [72]	*in vivo*	Charcoal meal passage rate (cm from cecum, NMRI-Haan mice, *in vivo*)	N/A	Unknown, oral in water	**Charcoal meal distance from cecum (cm):** Control: 10.85 ± 1.63 Industrial Hemp: 26.5 ± 9.90	CBD ↓ the distance traveled by a charcoal meal Extraction method limitations, unknown dosing
Pagano et al., 2016 [73]	*in vivo*	Charcoal meal passage rate (transit % of total length SI, ICR mice)	Croton oil (CO)	5–60 mg/kg, oral gavage 1–10 mg/kg, i.p.	**Passage rate (transit %) CBD Botanical Drug Substance (CBD BDS):** CBD BDS (10∗ mg/kg, IP): significant reduction in transit % of healthy mice CO: Increased transit %∗ CBD BDS (1∗, 2.5∗∗∗, 5∗∗∗, 10∗∗∗ mg/kg, IP) + CO: significant reduction in transit % compared to CO alone CBD BDS (5∗∗ & 10∗∗∗ mg/kg, IP) + CO: significant reduction compared to control (no CO) CBD BDS (10∗∗∗, 30∗, 60∗∗ mg/kg, oral): significant reduction in transit % of healthy mice CBD BDS (5∗∗, 10∗∗∗, 30∗∗∗, 60∗∗∗ mg/kg, oral) + CO: significant reduction in transit % compared to CO alone CBD BDS (10∗∗, 30∗∗, 60∗∗∗ mg/kg, oral) + CO: significant reduction in transit % compared to control (no CO) **Passage rate (transit %) CBD:** CBD (5∗∗ & 10∗ mg/kg, IP) + CO: significant reduction in transit % (data reported in figure) CBD (5∗∗ mg/kg, oral) + CO: significant reduction in transit % (data reported in figure)	% Transit was calculated by the distance the charcoal traveled in the small intestine The CBD BDS was 63.9% CBD Croton oil (CO) ↑ transit % CBD BDS (i.p. and oral) ↓ transit % in both control mice and CO treated mice CBD (i.p. and oral) ↓ % transit in CO treated mice but not of healthy controls
Shook and Burks, 1989 [70]	*in vivo*	Radioactive marker passage rate (% inhibition of small intestine transit, ICR mice)	N/A	Dose not reported, i.v.	**% inhibition of small intestine (SI) transit** CBD had no effect of SI transit %, data reported in figure, exact values unknown	CBD did not affect SI transit %
Lin et al., 2011 [77]	*in vitro/ex vivo* & *in vivo*	Charcoal meal passage (C57/BL mice, % transit of SI) SI myoelectrical activity (frequency and amplitude, Sprague-Dawley rat jejunum) Spontaneous contraction response (% control, Sprague-Dawley rat ileum and colon*)* Membrane potential (mouse jejunum)	LPS	*In vivo:* 1 mg/kg, i.p. *In vitro:* 0.001–0.1 μM	**SI myoelectrical spiking activity (SA) (% of control frequency & amplitude), *in vivo*:** LPS + CBD: Frequency and amplitude significantly higher than LPS∗∗ **Passage rate (SI transit % of control), *in vivo:*** LPS: 74.2 ± 3.6%∗ Significantly decreased compared to control LPS + CBD: 124.5 ± 9.4%∗ Significantly increased compared to LPS **Spontaneous Contraction response (% of control), *in vitro*:** LPS (ileum): 59.6 ± 7.4∗∗ Significantly decreased compared to control LPS (colon): 151.1 ± 27.6∗ Significantly increased compared to control LPS + CBD (0.001 μM∗, 0.01 μM∗∗, 0.1 μM∗∗ mol/L): Significantly increased % contraction compared to LPS group in both the ileum and colon tissue **Membrane potential (MP), *in vitro*:** CBD did not significantly affect the MP or amplitude of slow waves.	LPS ↓ frequency and amplitude of SA CBD pretreatment ↑ frequency and amplitude of SA in LPS treated rats LPS ↓ charcoal meal transit % CBD pretreatment ↑ meal transit % in LPS treated mice CBD normalized spontaneous contraction response in mouse ileum and colon segments CBD did not affect membrane potential
Capasso et al., 2008 [74]	*In vitro* & *in vivo*	Rhodamine-B-labeled dextran (geometric center of SI, ICR mice) Contractile response (ICR mouse terminal ileum*)*	*In vivo:* Croton oil, SR144528, naloxone, yohimbine, AA-5-HT, loperamide *In vitro:* ACh	*In vivo:* 1–10 mg/kg, i.p. *In vitro:* 0.01–100 μM	**Geometric center (GC) score of 1-10, *in vivo*:** Control (no croton oil): 4.91 ± 0.43 Croton oil (CO): 6.65 ± 0.41 (*P* < 0.05 vs control) CO + CBD (5 mg/kg): 5.01 ± 0.36∗ CO + CBD (5 mg/kg) + CB2 antagonist: 4.99 ± 0.38∗ CO + CBD (5 mg/kg) + naloxone: 4.98 ± 0.44∗ CO + CBD (5 mg/kg) + yohimbine: 4.97 ± 0.43∗ CO + CBD (5 mg/kg) + AA-5-HT: significantly decreased GC (values not shown) CO + CBD (10 mg/kg): significantly decreased the GC **Contractile response (% inhibition), *in vitro*:** Significantly decreased ACh induced contractions in control and croton oil treated tissue, however not significantly different from one another	GC was calculated as Σ (fraction of fluorescence per segment x segment number) Croton oil ↑ the geometric center CBD ↓ the GC of croton oil treated mice A CB2 antagonist, opioid receptor antagonist, and ⍺2-adrenoceptor antagonist did not modify the effect of CBD CBD did not have an additive effect when administered with a FAAH inhibitor CBD reduced ACh induced contractions in control and croton oil treated mouse ileum segments, however, the control and croton oil tissue % inhibition did not significantly differ from one another. CBD effected only croton oil treated mice *in vivo* but both control and treated mice *in vitro*
Wei et al., 2020 [78]	*in vitro/ex vivo* & *in vivo*	Charcoal passage (% of control, Sprague-Dawley rat) Disease activity index score (C57/BL mice) Contraction response to EFS (% if control, Sprague-Dawley rat colon) Membrane potential (colon, Sprague-Dawley rat colon)	TNBS	*In vivo:* 1 mg/kg, i.p. *In vitro:* 0.1 μM	**Charcoal passage (% of control), *in vivo*:** TNBS: Decreased compared to control∗∗ TNBS + CBD: Increased compared to TNBS∗∗ **Disease activity index (DAI) score, *in vivo*:** TNBS: Increased DAI significantly compared to control∗∗ TNBS + CBD: Decreased DAI compared to TNBS∗∗ **EFS contraction response (% of control), *in vitro*:** TNBS significantly increased contraction∗∗ CBD alone increased contraction∗ CBD decreased contraction compared to TNBS∗∗ **Membrane potential, *in vitro*:** CBD did not affect membrane potential. CBD did not alter the impact of TNBS on membrane potential.	TNBS ↓ charcoal meal passage % CBD had no effect on control mice charcoal meal passage % CBD ↑ the charcoal meal passage % in TNBS treated mice DAI score included weight loss, diarrhea, & bleeding. TNBS ↑ the DAI CBD ↓ the DAI of TNBS treated rats TNBS ↑ contraction response *in vitro* CBD blocked the effect of TNBS on EFS induced contractions CBD exerted an effect on EFS contractions of control tissue
Schicho et al., 2012 [81]	*in vivo*	Macroscopic assessment (change in score, CD1 mice, *in vivo*)	TNBS	10 mg/kg, i.p. 20 mg/kg, i.g. (intragastric) & i.r. (intrarectal)	**Macroscopic scoring:** TBNS + CBD, i.p.: significant reduction∗ TNBS + CBD, i.g.: not significant TNBS + CBD, i.r.: significant reduction∗	Diarrhea was one for seven variables in the macroscopic scoring. i.p. and i.r. administered CBD ↓ macroscopic score significantly
Becker et al., 2021 [82]	*in vivo*	Murine endoscopic index of colitis severity (change in score, C57BL/6 mice) Stool score (change in score, C57BL/6 mice)	DSS TNBS	10 mg/kg, oral gavage	**Murine endoscopic index of colitis severity (MEICS):** TNBS: increased score TNBS + CBD: no significant change TNBS + CBD + THC (10 mg/kg): significant reduction∗∗∗∗ **Stool Score:** DSS: increased stool score DSS + CBD: no significant change DSS + CBD + THC (10 mg/kg): significant reduction∗∗∗∗	Stool consistency was one for the four variables of the MEICS TNBS ↑ MEICS score CBD did not attenuate TNBS or DSS induced colitis scores CBD + THC ↓ MEICS and Stool Score THC was as effective alone as in combination with CBD
Yekhtin et al., 2022 [79]	*in vivo*	Clinical score (change in score, C57BL/6 mice)	DSS THC THC-extract	CBD, 5 mg/kg, i.p. every other day for 10 days CBD-extract (36% CBD, 1.3% THC), 5 mg/kg, i.p. every other day for 10 days	Difference between DSS group and all cannabinoid treatment groups∗∗∗ Difference between purified CBD and extract∗∗∗ Difference between purified CBD and purified THC∗	Clinical score was calculated from: stool score, rectal score, and general clinical parameters Significant reduction in clinical score with both purified CBD and CBD-extract CBD-extract decreased the clinical score more significantly than the purified CBD Purified THC decreased the score more significantly than purified CBD
Silvestri et al., 2020 [83]	*in vivo*	Disease Activity Index (change in score, CD1 mice, *in vivo*)	DSS Fish oil (FO)	0.3–10 mg/kg, oral gavage	**Disease Activity Index (DAI):** DSS + FO + CBD (1 mg/kg): significant reduction compared to DSS treated mice∗	The DAI score was assessed by stool consistency and blood in stool CBD alone did not affect the DAI CBD + fish oil ↓ DAI CBD activity followed a bell-shaped curve
Fride et al., 2005 [80]	*in vivo*	Defecation rate (maximal possible effect %, Sabra mice)	NA	20 mg/kg, i.p.	% Maximal possible effect (MPE) = Vehicle-Experiment/Vehicle x 100: CBD: 0%	CBD did not affect defecation rate in mice
Naftali et al., 2017 [85]	Human parallel group RCT	Crohn’s disease activity index (change in score)	N/A	20 mg/day for 8 weeks, sublingual (olive oil)	**Crohn’s disease activity index (CDAI) Score:** CBD treatment group (after treatment): 220 ± 122 Placebo treatment group (after treatment): 216 ± 121	CDAI score had 2 of 8 variables relating to defecation patterns Reduction in CDAI score was not significant Side effects/adverse effects did not differ between CBD and placebo group
Irving et al., 2018 [84]	Human parallel group RCT	Mayo score (total and partial score)	N/A	Up to 500 mg/day for 10 weeks (2 weeks escalation, 8 weeks maintenance), oral (gelatin capsule)	**Total Mayo score:** CBD botanical extract: decrease from baseline, however not significant **Partial Mayo Score:** CBD botanical extract: Significant decrease from baseline∗	Total mayo score includes stool frequency, rectal bleeding, endoscopy assessment, physician rating of disease activity; partial mayo score does not include endoscopy assessment The mean daily dose the CBD botanical extract was approximately 300 mg/day The CBD botanical extract group took fewer capsules and had more protocol compliance deviations than the placebo group The CBD botanical extract group had a ↑ % of AEs compared to the placebo group, the majority were mild to moderate CBD botanical extract ↓ partial mayo score significantly CBD botanical extract ↑ IBD quality of life assessment but was not statistically significant
Naftali et al., 2021 [86]	Human parallel group RCT	Bowel movement per day (change in number)	N/A	80 mg/day for 8 weeks, sublingual (cannabis oil)	**Bowel movements per day:** CBD (visit 3): 2.5 Placebo (visit 3): 3 No significant differences in bowel movements/day between groups	The CBD extract contained 16% CBD and 4% THC CBD did not significantly alter bowel movements per day compared to the placebo group No significant adverse effects were found in the CBD group compared to the placebo group
Van Orten-Luiten et al., 2021 [87]	Human crossover RCT	Changes in defecation patterns & IBS Quality of Life survey (change in score)	N/A	Up to 300 mg/day for 3 weeks, oral (chewing gum)	**QOL survey score:** Mean difference in Quality-of-Life score (CBD compared to placebo): 1.0 (*P* = 0.74)	Two 2 weeks intervention periods Chewing gum was associated with adverse effects CBD was not associated with any significant changes to Quality-of-Life score or defecation patterns

Abbreviations: AA-5-HT, arachidonoyl serotonin; ACh, acetylcholine; AE, adverse effects; CBD BDS, cannabidiol botanical drug substance; CBD, Cannabidiol; CDAI, Crohn’s disease activity index; CO, croton oil; DAI, disease activity index; DSS, dextran sulfate sodium; EFS, electrical field stimulation; FAAH, fatty acid amide hydrolase; FO, fish oil; GC, geometric center; GE, gastric emptying; IBD, inflammatory bowel disease; IBS, irritable bowel syndrome; KCl, potassium chloride; LPS, lipopolysaccharide; MEICS, murine endoscopic index of colitis severity; N/A, not applicable; RCT, randomized controlled trial; SA, piking activity; SI, small intestine; THC, tetrahydrocannabinol; TNBS, trinitrobenzene sulfonic acid; TTX, Tetrodotoxin; tx, treatment. ∗*P* < 0.05, ∗∗*P* < 0.01, ∗∗∗*P* < 0.001, *P* < 0.0001.

### *in vivo* studies investigating the effect of CBD on intestinal motility

#### Meal passage, meal transit, and geometric center measure distance and relative speed of travel through the intestines

One of the first studies investigating the effect of CBD on intestinal motility was performed in 1973 by Chesher et al. [69]. Motility was assessed by measuring charcoal meal passage rates in mice. Animals were sacrificed 15 min after charcoal meal administration. Purified CBD (6–30 mg/kg; oral delivery) did not significantly affect passage rates, though a full spectrum cannabis extract reduced transit time [69]. In a 1989 study, similar results were obtained when CBD was delivered using intravenous administration, without a discernible effect on intestinal transit of a radioactive marker [70]. The findings of these studies contradict a 1974 study which observed that orally delivered CBD reduced intestinal transit of charcoal meal in mice (10 mg/kg; oral delivery). When additional doses up to 50 mg/kg CBD were tested no effect was found, indicating a bell-shaped dose-response curve [71]. The oral administration vehicle of both Chesher et al. [69] (*n* = unreported) and Anderson et al. [71] (*n* = 15-50 per condition) was lissapol dispersal. Both studies used the same protocol for cannabidiol and charcoal meal administration and assessment of charcoal meal passage (percentage of small intestine traveled); however, the mice strains and sex did differ, which may have contributed to the varying results. In addition, the sample size used in the 1973 study was unreported.

In 2013, a study investigated the effect of high THC and high CBD isolates from hemp flower extracted by boiling water. The cannabinoid content of the extracts was not reported, and the mice were provided with the extract ad libitum [72]. It was found that a charcoal meal marker traveled significantly less distance through the intestines when mice were provided with the high CBD hemp extract compared to the control, indicating CBD may slow intestinal transit. Study limitations include the extraction method, as the lipid-soluble CBD is not readily extracted by water unless pressurized, and unknown dosing must be considered when interpreting these results [72].

Intestinal transit alterations following croton oil-induced hypermotility have been attenuated by CBD in multiple studies [73,74]. Capasso et al. [74] reported that CBD (5, 10 mg/kg; intraperitoneal) reduced the geometric center of a rhodamine-B-labeled dextran solution in croton oil-treated mice. Pagano et al. [73] reported that CBD has a protective effect against croton oil-induced hypermotility. The study compared the effects of isolated CBD to a CBD-rich *Cannabis sativa* extract (63.9% CBD, 3% THC) and reported that both the CBD-rich extract (1–10 mg/kg; intraperitoneal and 5–60 mg/kg; oral) and isolated CBD (5–10 mg/kg; intraperitoneal and 5 mg/kg; oral) significantly reduced the percent transit of a charcoal meal in croton oil-treated mice when provided orally and intraperitoneally delivered CBD at the doses denoted in the respective parenthesis [73]. The CBD-rich extract also significantly reduced the percent transit in healthy controls (10 mg/kg; intraperitoneal and 10–60 mg/kg; oral). Of note, isolated CBD (1–10 mg/kg; intraperitoneal and 5-60 mg/kg; oral) did not affect the percent transit in healthy mice at any dose [73]. The lack of CBD impact to healthy control groups is a phenomenon that has been demonstrated consistently *in vivo* [[74], [75], [76], [77], [78]].

The use of a high CBD cannabis extract was also studied in a murine dextran sulfate sodium (DSS)-induced colitis model of intestinal hypermotility [79]. CBD and a CBD-rich *C. sativa* extract (36% CBD, 1.3% THC) improved colitis presentation, which used a stool score, rated on a scale of 0 to 3, as one of three criteria integrated within the clinical score. Interestingly, and in agreement with Pagano et al. [73], the CBD-rich extract was more effective than the purified CBD.

In addition to having an effect in a hypermobile state, CBD affects intestinal function in a hypomobile state. In an experimental model of a septic ileus, De Filippis et al. [75] reported that CBD (10 mg/kg; intraperitoneal) administration to lipopolysaccharide (LPS)-treated mice significantly decreased the geometric center of orally administered glass beads. The CBD-associated reduction in geometric center was significant when compared both to healthy controls and to LPS-treated mice. These findings were contradicted by another report of CBD attenuating the effects of LPS treatment in mice rather than exacerbating LPS-induced hypomotility [77]. The study used a charcoal meal to measure the percent transit through the murine small intestine. It was found that LPS significantly reduced the percent transit and CBD administration (1 mg/kg, intraperitoneal) counteracted this effect [77]. The CBD administration methods and intestinal transit measurements differed between the 2 studies, which may explain some of the contradictory results. Recently, Wei et al. [78] reported that CBD counteracted trinitrobenzene sulfonic acid (TNBS)-induced hypomotility when using the same dose and administration method as Lin et al. [77] (1 mg/kg; intraperitoneal).

CBD has also been found to counteract the effects of synthetic cannabinoids, which themselves impact intestinal motility. Li et al. [76] reported that the synthetic cannabinoids O-1602 and WIN55,212-2 significantly increased whole gut transit of a marker solution in mice. CBD (0.5 mg/kg; intraperitoneal) significantly countered the effect of O-1602 but not that of WIN55,212-2. CBD (20 μg, intracerebroventricular) counteracted the effects of both O-1602 and WIN55,212-2 on whole gut transit [76].

#### Defecation patterns and stool consistency as a measure of intestinal motility

In 2005, Fride et al. [80] assessed the effect of CBD (20 mg/kg; intraperitoneal) on the defecation rate of mice and reported CBD had no effect. Li et al. [76] investigated the effects of synthetic cannabinoids on the rate of colonic bead expulsion in mice. The authors reported CBD itself did not affect bead expulsion, although CBD (0.5 mg/kg; intraperitoneal) did block the effects of O-1602.

Schicho and Storr [81] used a macroscopic scoring system that included diarrhea as 1 of 7 variables for mice with TNBS-induced colitis. CBD administered intraperitoneally (10 mg/kg) and intrarectally (20 mg/kg) significantly reduced the macroscopic colitis score; however, intragastric (20 mg/kg) administration did not have a significant effect. Wei et al. [78] also reported that CBD (1 mg/kg; intraperitoneal) reduced the effects of TNBS in mice using a scoring system. The disease activity score included measures of body weight loss, diarrhea, and bleeding and was significantly lower in animals pretreated with CBD (1 mg/kg; intraperitoneal). Becker et al. [82] used a murine endoscopic index of colitis severity (MEICS) and stool score to evaluate orally administered CBD on TNBS and DSS-induced colitis. Stool consistency was 1 of 4 variables included in the MEICS. The findings agreed with those of Schicho and Storr [81] in that orally delivered CBD (10 mg/kg) was ineffective at counteracting the effect of TNBS on the MEICS or DSS on the stool score. CBD (10 mg/kg; oral) did have a significant effect when administered with THC (10 mg/kg; oral), although THC alone exhibited similar efficacy [82]. Silvestri et al. [83] reported that orally delivered CBD (0.3–10 mg/kg) lacked an effect on the disease activity index, which was assessed using a diarrhea stool score and bloody stool score in DSS-treated mice. CBD in combination with fish oil, however, significantly reduced the disease activity index. Interestingly, fish oil alone did not significantly reduce the disease activity index to suggest a synergistic effect.

### Limited human studies have not yielded conclusive impact of CBD on intestinal motility

Four studies, with relatively small sample sizes (*n* = 19–62), have investigated the impact of CBD on gastrointestinal motility in human participants, and only one found a statistically significant improvement in aberrant motility measurements. Irving et al. [84] reported that CBD-rich *C. sativa* extract (≤500 mg/d, mean 300 mg/d, × 10 wk) improved the partial Mayo score of participants with ulcerative colitis. The partial Mayo score included stool frequency, rectal bleeding, and a physician global assessment of illness severity.

Two studies have investigated the impact of sublingual CBD supplementation in patients with Crohn’s disease. Neither found an impact of CBD on the Crohn’s disease activity index (20 mg/d × 8 wk) or the number of bowel movements per day (80 mg/d × 8 wk) [85,86].

Participants with diarrhea-predominant IBS were provided with up to 300 mg/d CBD for 2 weeks in chewing gum. CBD was not associated with any significant effects or changes in gastrointestinal function including defecation patterns. Challenges using CBD chewing gum were noted, including participant adherence to chewing time guidelines (30 min), number of doses/gums taken, and reports of unpleasant air ingestion rendering the delivery method potentially ineffective [87].

### *in vitro* and *ex vivo* studies of CBD impact on intestinal motility

*in vitro* and *ex vivo* studies investigate the role of CBD in regulating gastrointestinal motility to advance preclinical and inform clinical research. It is widely acknowledged that these models lack the complexity of *in vivo* studies, although human research has been restricted by the evolving regulatory posture on cannabinoid research.

#### Electrical field stimulation assesses the effect of CBD on contractile responses

Gastrointestinal tract muscle contractions are ultimately what controls intestinal motility. Electrical field stimulation (EFS) is a method to quantify the impact of exogenous compounds on the contractile response. Accordingly, Cluny et al. [88] reported that CBD (10^-5^ M) significantly reduced EFS-induced contraction in proximal and central intestine tissue of *Suncus murinus* at high frequencies of stimulation (4–20 Hz) but not low frequencies*.* The varied responses to different frequencies suggest CBD influences motility during specific myoelectrical activity patterns, although this remains speculative. CBD did not modify the response induced by EFS in *S. murinus* terminal intestine tissue [88,89]. The effect of CBD (10^-7^ M) on specific parts of intestinal tissue was demonstrated to reduce EFS-induced contractions in the murine colon but not ileum [76].

CBD counteracts effects of TNBS-induced colitis in an organ bath model. More specifically, CBD (10^-7^ M) blocks contraction response to EFS (10 Hz) when treated with TNBS in the colon. CBD also significantly increased the contraction response in the absence of TNBS [78].

The effect of *in vivo* CBD treatment on *in vitro* measures of motility was assessed by Jamontt et al. [90]. CBD (5–20 mg/kg; intraperitoneal) did not affect EFS responses in intestinal segments in mice treated with TNBS (1–15 Hz); however, CBD/THC (both 10 mg/kg; intraperitoneal) significantly increased relaxation and contraction to EFS toward the control values [90]. An additive effect was demonstrated for CBD (10 mg/kg; intraperitoneal) and THC (5 mg/kg; intraperitoneal) and reached significance in the relaxant response at 15 Hz [90].

#### CBD varies in influence on chemically induced contractions

Carbachol mimics the effects of acetylcholine, which is a neurotransmitter that facilitates gastrointestinal contractions and motility [91]. The contraction response to carbachol was significantly reduced when CBD was applied to intestinal segments of the proximal (3 × 10^-6^ M and 10^-5^ M CBD), central (10^-5^ M CBD), and terminal (10^-5^ M CBD) segments of *S. murinus* [88]. Contradictory results were reported when mice were provided with CBD prior to sacrifice, rather than being directly applied to intestine segments postmortem. Jamontt et al. [90] reported that CBD had a beneficial effect on aberrations in carbachol response subsequent to TNBS treatment. CBD treatment counteracted the effects of TNBS treatment, which had decreased carbachol response in intestinal tissue. CBD (10 mg/kg; intraperitoneal) and CBD/THC (10 mg/kg; intraperitoneal each) both significantly increased the tissue response to carbachol. The combination of CBD/THC induced a greater response than THC (10 mg/kg) alone [90].

Capasso et al. [74] reported that CBD (10^-5^–0.1 M) itself had no effect on baseline tissue contractility. However, when acetylcholine was used to induce contractions, CBD significantly decreased contractions in mouse ileum segments in a dose-dependent manner in tissue from healthy mice and mice treated with croton oil, which was used to induce inflammation [74]. A similar lack of a contraction response to CBD in mouse ileum and colon tissue were reported using concentrations of 10^-9^ and 10^-8^ M (76). CBD (10^-8^–10^-5^ M) also failed to induce contractions in mouse jejunum tissue [75]. Contradictory results were reported by Cluny et al. [88], who found that CBD (10^-8^–3×10^-5^ M) reduced resting tissue contractions in all parts of *S. murinus* intestines in a dose-dependent manor with significance reached at >10^-6^ M CBD.

#### Spontaneous activity as a measure of anomalous contractility in intestine tissue

Spontaneous activity of tissue is a measure of the amplitude of contraction in the absence of stimulation. These unstimulated events may shed light on aberrations in contractile function, thus motility, and can be compared between treatments/conditions both in frequency and magnitude. CBD attenuated aberrations in spontaneous activity of animals treated with LPS and TNBS, both when CBD was applied to excised tissue and provided via intraperitoneal injection to the animals prior to sacrifice [77,90]. Application of CBD (10^-9^–10^-7^ M) directly to tissue normalized spontaneous contractions that had been disrupted by LPS in rat ileum and colon segments [77].

Segments with TNBS-induced colitis had a significant reduction in amplitude and increased duration of spontaneous contractions. CBD (10 mg/kg; intraperitoneal) and CBD/THC (both 10 mg/kg; intraperitoneal) significantly increased the amplitude. CBD (10 or 20 mg/kg; intraperitoneal) and varying relative concentrations of CBD/THC significantly reduced duration [90]. Membrane potential is also a measure of muscle tissue activity; however, two studies have reported CBD (10^-7^ M) does not significantly affect membrane potential, either mouse jejunum [77] or rat colon [78].

## Discussion

The effectiveness of CBD as a therapeutic intervention varies by condition, dosing, delivery method, and inflammatory status, along with other unidentified factors. To date, there is limited evidence for CBD ameliorating gastrointestinal symptoms of both IBS and IBD in humans [[84], [85], [86], [87]] based on the four human studies with vastly differing doses and administration routes identified during this systematic review. The dosing between these studies have varied from 20 to 500 mg/d of CBD. It has been proposed CBD has a bell-shaped activity curve, and it is possible that the active dose of CBD was not utilized in these studies [71,90,92].

Along with difficulty in determining an active dose, additional factors such as dietary intake, delivery route, and the specific formulation of CBD impacts its bioavailability. CBD is a hydrophobic molecule and has a high rate of phase 1 metabolism [25,93]. Currently, there is a knowledge gap on the biological activity of CBD metabolites [25]. One of the few studies on CBD derivatives reported that 7-COOH-CBD decreased defecation rates in mice [80]. Therefore, there is preliminary evidence that CBD metabolites could contribute to the global effects of CBD administration. Future research will define the role of CBD metabolites on human physiological function.

Delivery route, such as oral, sublingual, or inhalation, influences the concentration of cannabinoids that reach systemic circulation and avoid phase 1 metabolism. The oral bioavailability of CBD is comparatively low compared to smoking, which is between two to eight times higher [26]. Inhalation of vaporized CBD may lead to even higher bioavailability, although additional research is needed to assess methods of CBD administration using standardized metrics. Orally delivered CBD may have too low of a bioavailability to exert a measurable effect on disease activity scores in animal models [[81], [82], [83]]. Interestingly, when coadministered with fish oil, CBD was demonstrated to not only significantly improve disease activity but also markers of intestinal inflammation and intestinal barrier function to suggest a potential synergistic effect to be investigated in follow-up studies.

CBD has been demonstrated *in vitro* to have varying effects based on the specific location of the intestine into which it is introduced [76,88]. Moreover, CBD modulates intestinal motility in a contrasting manner between *in vitro* and *in vivo* models in the studies performed to date. Regardless, if this is due to the limited research in this developing field, these contrasting results limit the conclusions that can be applied to *in vivo* function from *in vitro* research [74,78]. Both Capasso 2008 and Wei 2020 reported contradicting results of CBD treatment between *in vitro* and *in vivo* intestinal motility models [74,78].

Large interindividual variations have hindered conclusions on the impact of CBD delivery methods [94]. Enhanced oral delivery systems, such as nanoemulsions, have been demonstrated to increase bioavailability [95] and could target specific anatomical sites of activity with customizable time release characteristics. Encapsulation platforms such as emulsions, liposomes, solid lipid nanoparticles, and microgels are also effective with chemically similar compounds [93].

Food intake, particularly dietary fat, has been demonstrated to increase peak plasma CBD concentrations (C_max_) and overall exposure as measured by AUC. Multiple studies have found the C_max_ and AUC of orally delivered CBD are higher when administered with food [21,96,97]. Excipient food products, that is, food with specific ingredients or qualities to enhance bioavailability, could also benefit CBD users by modifying the absorption characteristics [93]. Additional research will optimize dietary intake recommendations to enhance CBD pharmacokinetic and pharmacodynamic profiles.

An inherent challenge in designing delivery systems is the incomplete understanding of the mechanisms by which CBD influences physiological function. It is unclear if CBD exerts an effect through direct interaction with receptors or indirectly, such as regulation of inflammatory status. The anti-inflammatory characteristics of CBD have been extensively studied [8,98,99]. Of interest, the effect of CBD on intestinal motility may be mediated through reduction of inflammation as CBD attenuates intestinal inflammation, as assessed through a variety of inflammatory markers including: myeloperoxidase activity, inflammatory cytokines, reactive oxygen species production, histopathology, and nitric oxide production [78,79,100].

It is possible that CBD influences intestinal motility through the gut-brain axis, which is a bidirectional neural system that affects physiological functions throughout the human body [101]. Accordingly, the gut microbiome produces signaling molecules, including neurotransmitters, that participate in the gut-brain axis and therefore represent an additional therapeutic target to address intestinal and neurological disorders [30,101]. Few studies have investigated the impact of CBD on the gut microbiome, although enrichment of beneficial bacteria and their products, such as short chain fatty acids, have been reported [83,102,103].

## Perspectives

CBD is a popular supplement in the United States, in addition to its use as a pharmaceutical drug. *in vitro* and animal model studies suggest the potential for CBD to alleviate motility disturbances; however, human studies have yet to characterize broadly significant effects. Establishing optimal delivery parameters is critical for standardization of research and, importantly, CBD applications to effectively intervene in human physiology, including gastrointestinal motility. Further research will deploy systems-level approaches to quantitatively link multifactorial processes with qualitative participant outcomes to generate a rigorous understanding of the therapeutic effects of CBD on gastrointestinal motility disorders. It is critical to mechanistically characterize any direct interactions between orally delivered CBD with endocannabinoid receptors in the gut as well as indirect interactions with the enteric nervous system. Furthermore, anti-inflammatory effects of CBD may influence gastrointestinal peristalsis via overlapping mechanisms as well as potentially select microbiota that propagate systemic effects to influence gut motility.

## Funding

This work was funded, in part, by the U.S. Department of Agriculture under Hatch Grant MAS00556 (DAS) and The College of Natural Sciences at the University of Massachusetts Amherst through a Bridge and Seed Grant (DAS). Partial fellowship support was provided by the Peter Salmon Graduate Fellowship (GS).

## Author contributions

The authors’ responsibilities were as follows – DAS: conceived research; DAS, GS: designed research; GS: conducted research, extracted data, and prepared the first draft of the manuscript; GS, DAS: conducted the selection and evaluation of studies; and all authors: read and approved the final manuscript.

## Conflict of interest

DJM is currently on the advisory board for Vertosa, which manufacturers CBD products. Vertosa did not have any role in preparing or reviewing this manuscript. All other authors report no conflicts of interest.
