# Supplementary material for: Cannabidiol and Intestinal Motility: a Systematic Review
Source: Curr Dev Nutr. 2023 Jul 17;7(10):101972. doi: 10.1016/j.cdnut.2023.101972 (PMC10541995; doi:10.1016/j.cdnut.2023.101972)
Supplement: Multimedia component 1 [file mmc1.docx]

**Cannabidiol and intestinal motility: A Systematic Review**

Galaxie Story^1^, Carrie-Ellen Briere^2^, D. Julian McClements^1^, and David A. Sela^1,3,4*^

^1^ Department of Food Science, University of Massachusetts, Amherst, Massachusetts 01003, United States

^2^ Elaine Marieb College of Nursing, University of Massachusetts, Amherst, Massachusetts 01003, United States

^3^ Department of Nutrition, University of Massachusetts, Amherst, Massachusetts 01003, United States

^4^ Department of Microbiology and Physiological Systems, University of Massachusetts Medical School, Worcester, Massachusetts 01655, United States

*Corresponding author: David A. Sela

E-mail address: davidsela@umass.edu

Phone number: 413-545-1010

Running title: **Cannabidiol and intestinal motility**

**PUBMED SEARCHES: 12/21/2021**

**Updated 12/07/2022**

(cannabidiol) AND motility

(((cbd) AND cannabidiol) AND IBD) AND inflammatory bowel disease

(cannabidiol) AND inflammatory bowel disease

(cannabidiol) AND intestinal inflammation

((((cannabidiol) AND inflammation) AND cbd) AND bowel) AND colitis

(cannabidiol) AND colitis

(cannabidiol) AND gut

(cannabidiol) AND intestinal

(cannabidiol) AND intestine

(cannabidiol) AND colon

cannabinoid Inflammatory bowel disease

(cannabinoid) AND colitis

(cannabinoid) AND gut microbiota

(cannabinoid) AND gut microbiome

(cannabidiol) AND microbiome

(cannabidiol) AND gastrointestinal

(cannabidiol) AND gastroenterology

**Clinical Trails Gov US:**

Terms and Synonyms Searched:

Terms Search Results* Entire Database**

“Cannabidiol”
